# Supplementary figures and images for: Tissue-specific inhibition of protein sumoylation uncovers diverse SUMO functions during C. elegans vulval development
Source: PLoS Genet. 2022 Jun 6;18(6):e1009978. doi: 10.1371/journal.pgen.1009978 (PMC9203017; doi:10.1371/journal.pgen.1009978)

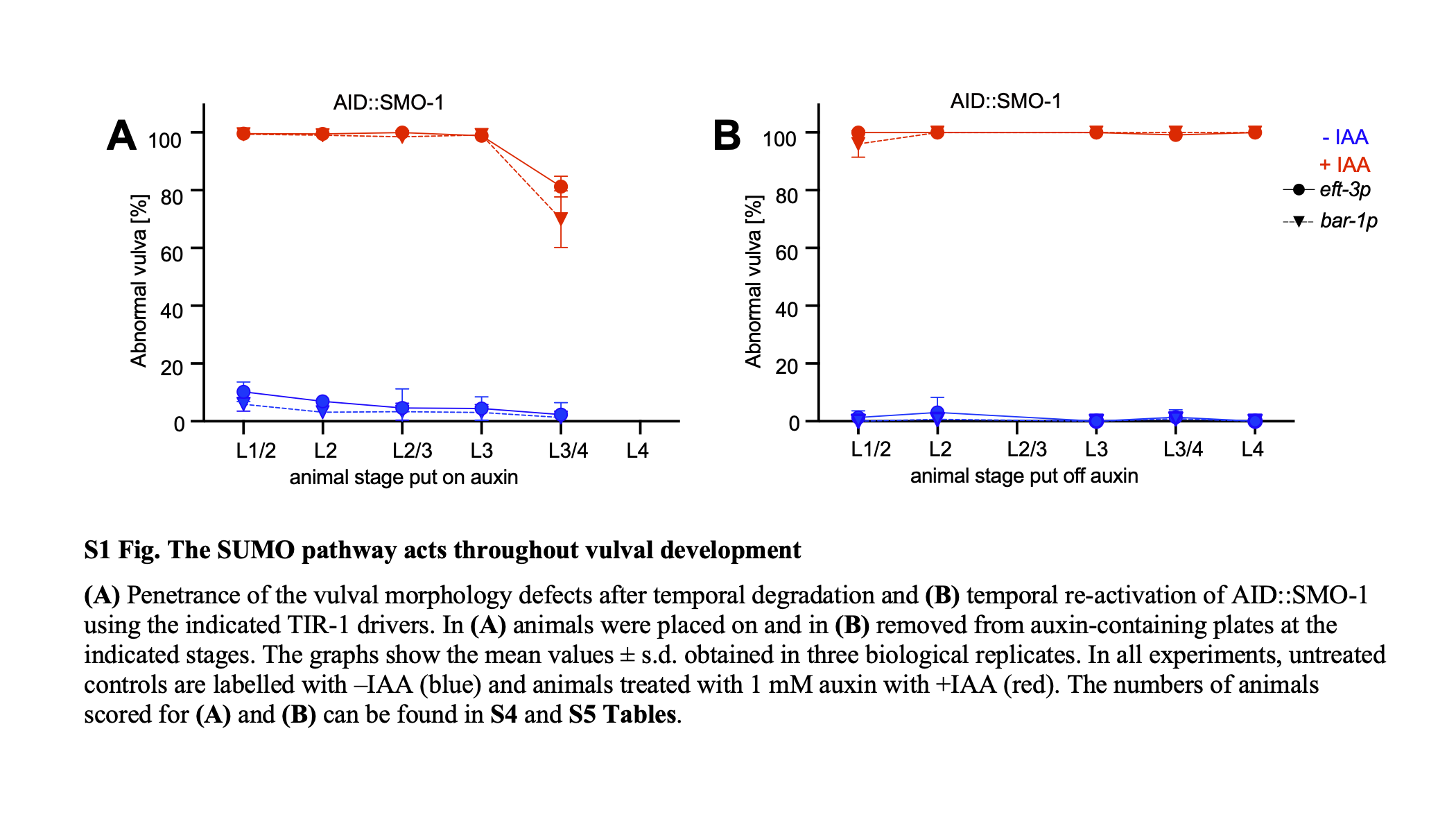

Supplement: S1 Fig — (TIFF) [file pgen.1009978.s001.tiff]

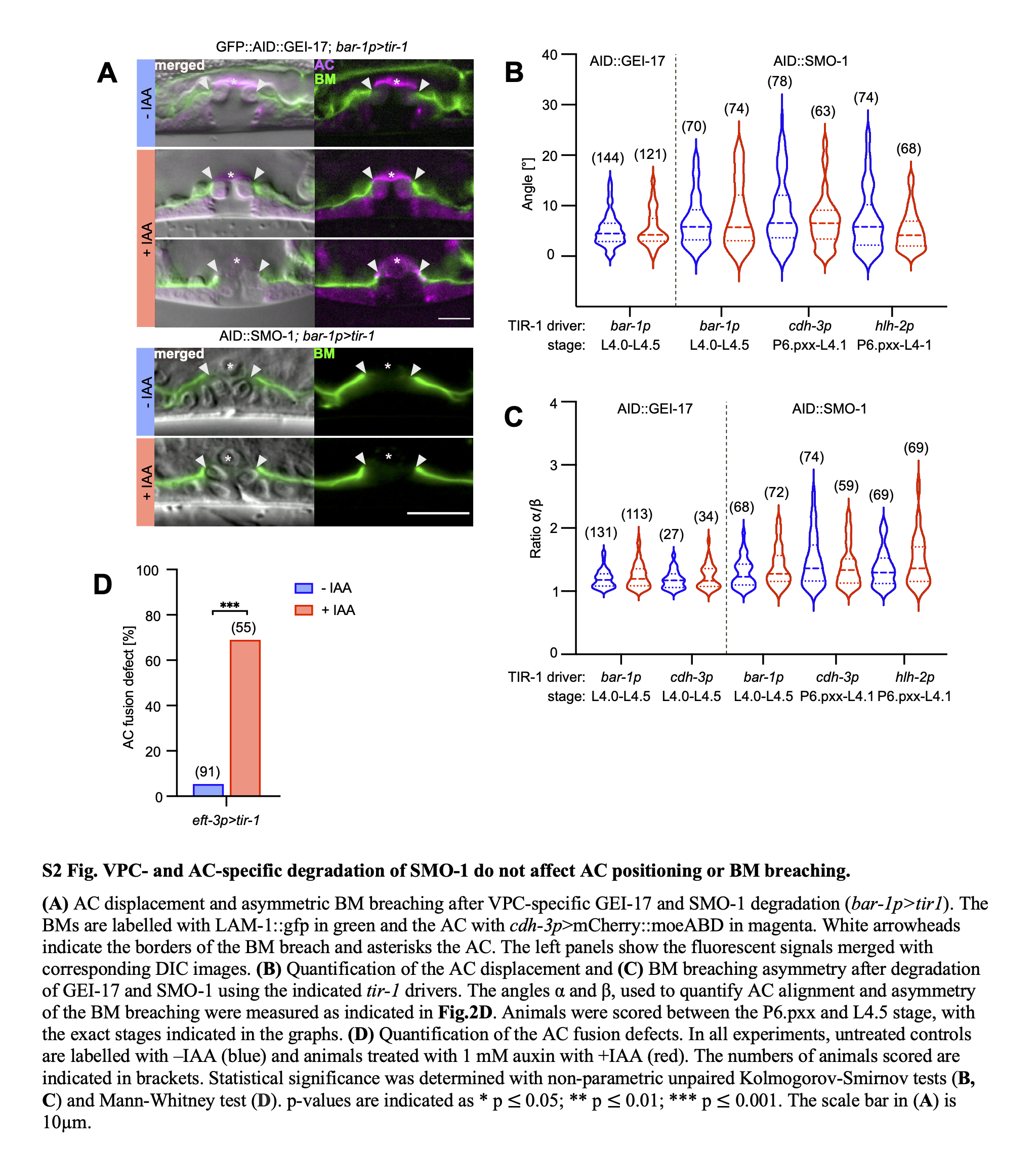

Supplement: S2 Fig — (TIFF) [file pgen.1009978.s002.tiff]

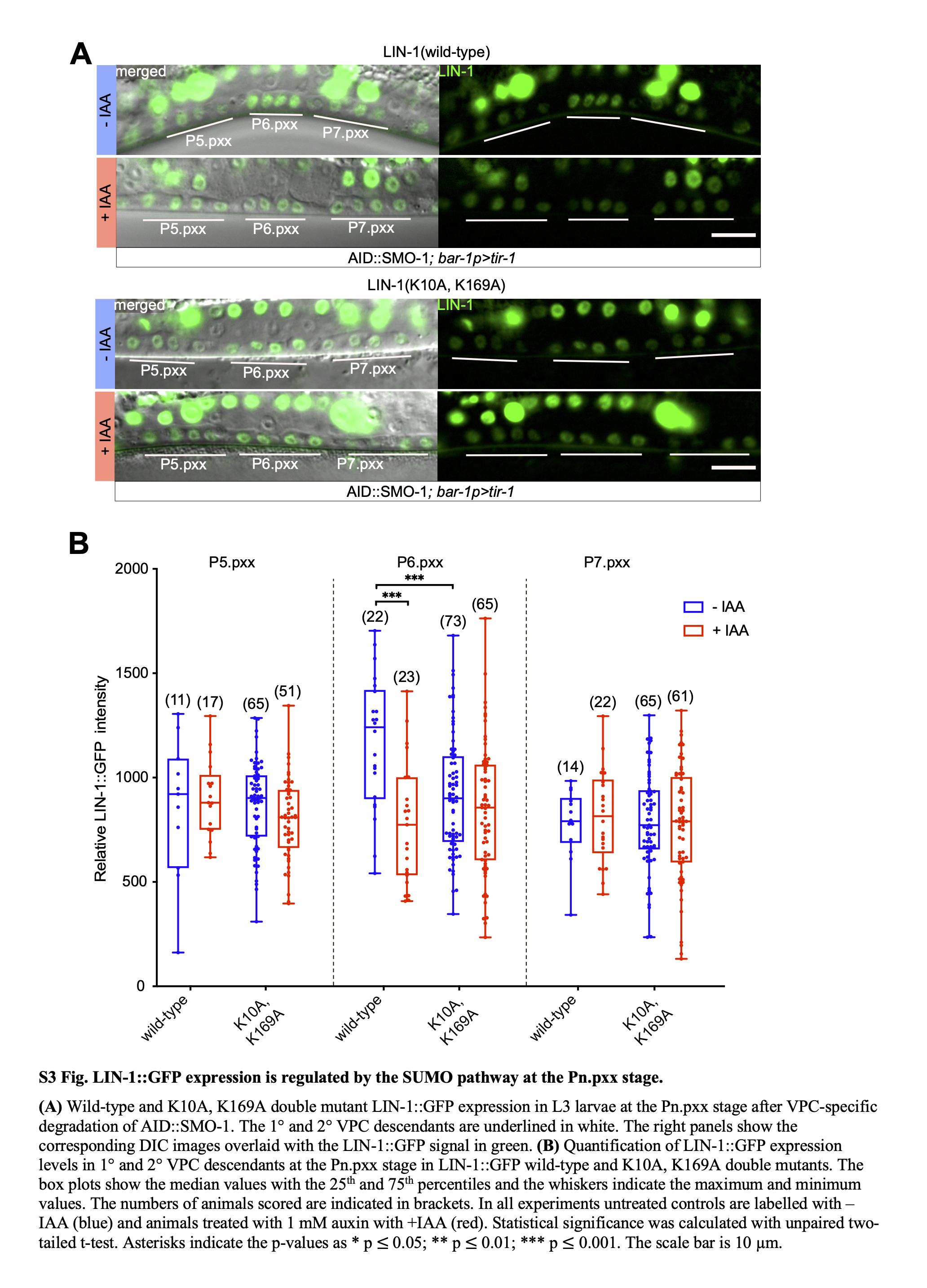

Supplement: S3 Fig — (TIFF) [file pgen.1009978.s003.tiff]
