# Supplementary material for: Tissue-specific inhibition of protein sumoylation uncovers diverse SUMO functions during C. elegans vulval development
Source: PLoS Genet. 2022 Jun 6;18(6):e1009978. doi: 10.1371/journal.pgen.1009978 (PMC9203017; doi:10.1371/journal.pgen.1009978)
Supplement: S1 Table — (DOCX) [file pgen.1009978.s004.docx]

## S1 Table. Strains used

| Strain | Genotype | Reference |
| --- | --- | --- |
| AH3420 | *smo-1(ok359)/ht2[bli-4(e937) let-?(q782) qIs48] (I; III)* | this study |
| AH5112 | *gei-17(fgp1[gfp::FLAG::AID::loxP::gei-17]) I* | CGC* strain FGP30 [1] outcrossed with N2 |
| AH5129 | *zhIs123[egl-17p>TIR-1::SL2::mCherry::unc-54 3`UTR, unc-119 (+)] II; unc-119(ed3) III* | this study |
| AH5141 | *gei-17(fgp1[gfp::FLAG::AID::loxP::gei-17]) I; ieSi57 [eft-3p>TIR-1::mRuby::unc-54 3'UTR, Cbr-unc-119(+)] II; unc-119(ed3) III* | this study |
| AH5168 | *gei-17(fgp1[gfp::FLAG::AID::loxP::gei-17]) I; zhIs123[egl-17p>TIR-1::SL2::mCherry::unc-54 3`UTR, unc-119 (+)] II; unc-119(ed3) III; qyIs10[lam-1::gfp; unc-119(+)] IV; him-5(e1490)/+ V* | this study |
| AH5169 | *gei-17(fgp1[gfp::FLAG::AID::loxP::gei-17]) I; ieSi57 [eft-3p>TIR-1::mRuby::unc-54 3'UTR, Cbr-unc-119(+)] II; unc-119(ed3) III; qyIs10[lam-1::gfp; unc-119(+)] IV; him-5(e1490)/+ V* | this study |
| AH5525 | *gei-17(fgp1[gfp::FLAG::AID::loxP::gei-17]) I; ieSi57 [eft-3p>TIR-1::mRuby::unc-54 3'UTR, Cbr-unc-119(+)] II; unc-119(ed3) III; qyIs10[lam-1::gfp; unc-119(+)] IV; him-5(e1490)/+ V; qyIs50[cdh-3>mCherry::moeABD; unc-119(+)] V* | this study |
| AH5527 | *gei-17(fgp1[gfp::FLAG::AID::loxP::gei-17]) I; zhIs129[bar-1p>TIR-1::SL2::mCherry::unc-54 3` UTR, unc-119 (+)] II; unc-119(ed3) III; qyIs10[lam-1::gfp; unc-119(+)] IV; him-5(e1490)/+ V; qyIs50[cdh-3>mCherry::moeABD; unc-119(+)] V* | this study |
| AH5547 | *zhIs129[bar-1p>TIR-1::SL2::mCherry::unc-54 3`UTR, unc-119 (+)] II; unc-119(ed3) III* | this study |
| AH5554 | *gei-17(fgp1[gfp::FLAG::AID::loxP::gei-17]) I; zhIs129[bar-1p>TIR-1::SL2::mCherry::unc-54 3` UTR, unc-119 (+)] II; unc-119(ed3) III; qyIs10[lam-1::gfp; unc-119(+)] IV; him-5(e1490)/+ V* | this study |
| AH5587 | *gei-17(fgp1[gfp::FLAG::AID::loxP::gei-17]) I; zhIs129[bar-1p>TIR-1::SL2::mCherry::unc-54 3` UTR, unc-119 (+)] II; unc-119(ed3) III; qyIs10[lam-1::gfp; unc-119(+)] IV; him-5(e1490)/+ V; qyIs50[cdh-3>mCherry::moeABD; unc-119(+)] V* | this study |
| AH5609 | *zhIs133[cdh-3p>TIR-1::SL2::mCherry::unc-54 3`UTR, unc-119 (+)] II; unc-119(ed3) III* | this study |
| AH5619 | *gei-17(fgp1[gfp::FLAG::AID::loxP::gei-17]) I; zhIs133[cdh-3p>TIR-1::SL2::mCherry::unc-54 3`UTR, unc-119 (+)] II; unc-119(ed3) III; qyIs10[lam-1::gfp; unc-119(+)] IV; him-5(e1490)/+ V* | this study |
| AH5670 | *gei-17(zh142[degron::loxP::3xFLAG::gei-17]) I; zhIs129[Pbar-1::TIR1::SL2::mCherry::unc-54 3` UTR, unc-119 (+)] II; unc-119(ed3) III* | this study |
| AH5673 | *gei-17(fgp1[gfp::FLAG::AID::loxP::gei-17]) I; zhIs129[bar-1p>TIR-1::SL2::mCherry::unc-54 3` UTR, unc-119 (+)] II; unc-119(ed3) III; zhIs138[cdh-3p>TIR-1::SL2::mCherry::unc-54 3`UTR, unc-119 (+)] III; unc-119(ed3) III; qyIs10[lam-1::gfp; unc-119(+)] IV; him-5(e1490)/+ V* | this study |
| AH5675 | *gei-17(fgp1[gfp::FLAG::AID::loxP::gei-17]) I; zhIs123[egl-17p>TIR-1::SL2::mCherry::unc-54 3`UTR, unc-119 (+)] II; unc-119(ed3) III; zhIs138[cdh-3p>TIR-1::SL2::mCherry::unc-54 3`UTR, unc-119 (+)] III; unc-119(ed3) III]; qyIs10[lam-1::gfp; unc-119(+)] IV; him-5(e1490)/+ V* | this study |
| AH5685 | *gei-17(zh142[AID::loxP::FLAG::gei-17]) I; ieSi57 [bar-1p>TIR-1::mRuby::unc-54 3'UTR, Cbr-unc-119(+)] II; unc-119(ed3) III; swIs79[ajm-1::gfp, seam cell::gfp, unc-119(+)] IV* | this study |
| AH5711 | *smo-1(zh140[AID::loxP::3xFLAG::smo-1])/tmC20[unc-14(tmIs1219) dpy-5(tm9715)] I;* | this study |
| AH5731 | *smo-1(zh140[AID::loxP::3xFLAG::smo-1])/tmC20[unc-14(tmIs1219) dpy-5(tm9715)] I; ieSi57 [eft-3p>TIR-1::mRuby::unc-54 3'UTR, Cbr-unc-119(+)] II; unc-119(ed3) III* | this study |
| AH5736 | *gei-17(zh142[AID::FLAG::gei-17]) I; zhIs129[bar-1p>TIR-1-SL2-mCherry::unc-54 3`UTR, unc-119 (+)] II; unc-119(ed3) III; syIs90[egl-17::yfp, unc-119] III* | this study |
| AH5740 | *smo-1(zh140[AID::loxP::3xFLAG::smo-1])/tmC20[unc-14(tmIs1219) dpy-5(tm9715)] I; zhIs129[bar-1p>TIR-1::SL2::mCherry::unc-54 3` UTR, unc-119 (+)] II; unc-119(ed3) III* | this study |
| AH5802 | *smo-1(zh140[AID::loxP::3xFLAG::smo-1])/tmC20[unc-14(tmIs1219) dpy-5(tm9715)] I; zhIs123[egl-17p>TIR-1::SL2::mCherry::unc-54 3`UTR, unc-119 (+)] II; unc-119(ed3) III* | this study |
| AH5808 | *smo-1(zh140[AID::loxP::3xFLAG::smo-1])/tmC20[unc-14(tmIs1219) dpy-5(tm9715)] I; zhIs138[cdh-3p>TIR-1::SL2::mCherry::unc-54 3`UTR, unc-119 (+)] III; unc-119(ed3) III* | this study |
| AH5824 | *smo-1(zh140[degron::loxP::3xFLAG::smo-1]) I/tmC20 [unc-14(tmIs1219) dpy-5(tm9715)] I; zhIs138[Pcdh-3::TIR1::SL2::mCherry::unc-54 3`UTR, unc-119 (+)] III; unc-119(ed3) III; qyIs10[lam-1::gfp; unc-119(+)] IV; him-5(e1490) V* | this study |
| AH5869 | *hmr-1(cp21[hmr-1::gfp + LoxP]) I; smo-1(zh140[AID::loxP::3xFLAG::smo-1])/tmC20[unc-14(tmIs1219) dpy-5(tm9715)] I; zhIs129[bar-1p>TIR-1::SL2::mCherry::unc-54 3`UTR, unc-119 (+)] II; unc-119(ed3) III; qyIs50[cdh-3>mCherry::moeABD; unc-119(+)] V* | this study |
| AH5937 | *smo-1(zh140[AID::loxP::3xFLAG::smo-1])/tmC20[unc-14(tmIs1219) dpy-5(tm9715)] I; zhIs123[egl-17p >TIR-1::SL2::mCherry::unc-54 3`UTR, unc-119 (+)] II; unc-119(ed3) III; zhIs138[cdh-3p>TIR-1::SL2::mCherry::unc-54 3`UTR, unc-119 (+)] III* | this study |
| AH5953 | *smo-1(zh140[AID::loxP::3xFLAG::smo-1])/tmC20[unc-14(tmIs1219) dpy-5(tm9715)] I; zhIs129[bar-1p>TIR-1::SL2::mCherry::unc-54 3` UTR, unc-119 (+)] II; unc-119(ed3) III; lin-1(st12212[lin-1::TY1::egfp::3xFLAG]) IV* | this study |
| AH5997 | *zhIS147 [hlh-2prox>TIR-1::SL2::mCherry::unc-54 3`UTR + unc-119 (+)] III; unc-119(ed3) III* | this study |
| AH6031 | *smo-1(zh140[AID::loxP::3xFLAG::smo-1])/tmC20[unc-14(tmIs1219) dpy-5(tm9715)] I; zhIS147 [hlh-2prox>TIR-1::SL2::mCherry::unc-54 3`UTR, unc-119 (+)] III; unc-119(ed3) III* | this study |
| AH6047 | *smo-1(zh140[AID::loxP::3xFLAG::smo-1])/tmC20[unc-14(tmIs1219) dpy-5(tm9715)] I; ieSi57 [eft-3p>TIR-1::mRuby::unc-54 3'UTR, Cbr-unc-119(+)] II; unc-119(ed3) III; qyIs50[cdh-3>mCherry::moeABD; unc-119(+)] V; qyIs10[lam-1::gfp; unc-119(+)] IV; him-5(e1490)/+ V* | this study |
| AH6050 | *smo-1(zh140[AID::loxP::3xFLAG::smo-1])/tmC20[unc-14(tmIs1219) dpy-5(tm9715)] I; zhIs129[bar-1p>TIR-1::SL2::mCherry::unc-54 3` UTR, unc-119 (+)]II; unc-119(ed3) III; qyIs10[lam-1::gfp; unc-119(+)] IV; him-5(e1490)/+ V* | this study |
| AH6073 | *smo-1(zh140[AID::loxP::3xFLAG::smo-1])/tmC20[unc-14(tmIs1219) dpy-5(tm9715)] I; zhIs129[bar-1p>TIR-1::SL2::mCherry::unc-54 3` UTR, unc-119 (+)] II; unc-119(ed3) III; lin-1(zh159)[st12212[lin-1(K10A, K169A)::TY1::egfp::3xFLAG]) IV* | this study |
| AH6095 | *lin-1(zh157)[st12212[lin-1(K10A)::TY1::egfp::3xFLAG]) IV; hmr-1(cp21[hmr-1::gfp + LoxP]) I* | this study |
| AH6096 | *lin-1(zh158)[st12212[lin-1(K169A)::TY1::egfp::3xFLAG]) IV; hmr-1(cp21[hmr-1::gfp, LoxP]) I* | this study |
| AH6097 | *lin-1(zh159)[st12212[lin-1(K10A, K169A)::TY1::egfp::3xFLAG]) IV; hmr-1(cp21[hmr-1::gfp + LoxP]) I* | this study |
| AH6100 | *smo-1(zh140[degron::loxP::3xFLAG::smo-1]) I/tmC20 [unc-14(tmIs1219) dpy-5(tm9715)] I; zhIS147 [hlh-2prox::TIR1::SL2::mCherry::unc-54 3`UTR, unc-119(+)] III; unc-119(ed3) III; qyIs10[lam-1::gfp; unc-119(+)] IV; him-5(e1490) V* | this study |
| RW12212 | *lin-1(st12212[lin-1::TY1::egfp::3xFLAG]) IV* | CGC* |

**C. elegans* Genetics Center (Minneapolis, MN)

**Reference**

1. Pelisch F, Tammsalu T, Wang B, Jaffray EG, Gartner A, Hay RT. A SUMO-Dependent Protein Network Regulates Chromosome Congression during Oocyte Meiosis. Mol Cell. 2017;65: 66–77. doi:10.1016/j.molcel.2016.11.001
