# Supplementary material for: Tissue-specific inhibition of protein sumoylation uncovers diverse SUMO functions during C. elegans vulval development
Source: PLoS Genet. 2022 Jun 6;18(6):e1009978. doi: 10.1371/journal.pgen.1009978 (PMC9203017; doi:10.1371/journal.pgen.1009978)
Supplement: S2 Table — (DOCX) [file pgen.1009978.s005.docx]

**S2 Table List of plasmids used**

| pAF25 | sgRNA: GCCGATGATGCAGCTCAAGC targeting N-terminal of *smo-1* in pMW46 |
| --- | --- |
| pAF44 | sgRNA: GTCGTTTCGAGACACAGCGG targeting N-terminus of *gei-17* in pMW46 |
| pAF50 | pCFJ151 containing egl-17p>TIR-1::SL2::mCherry::unc-54 3`UTR |
| pAF52 | pCFJ151 containing bar-1p>TIR-1::SL2::mCherry::unc-54 3`UTR |
| pAF55 | pCFJ151 containing cdh-3p>TIR-1::SL2::mCherry::unc-54 3`UTR |
| pAF57 | repair template for AID::LoxP::SEC::LoxP::3xFlag::SMO-1 |
| pAF58 | repair template for AID::LoxP::SEC::LoxP::3xFlag::GEI-17 |
| pAF59 | sgRNA: GTC GAG TTC GGA AGA AGC CG targeting K10 in *lin-1* cloned in pMW46 |
| pAF62 | sgRNA: GTT CAT ATT TGA GGA AAA GT targeting K169 in *lin-1* cloned in pMW46 |
| pAF63 | pCFJ151 containing hlh-2prox>TIR-1::SL2::mCherry::unc-54 3`UTR |

**Details on the plasmid constructions**

pAF25 – sgRNA: GCC GAT GAT GCA GCT CAA GC targeting N-terminal of *smo-1*. Cloned with OAF173 (cctg GCC GAT GAT GCA GCT CAA GC) and OA174 (aaac GCT TGA GCT GCA TCA TCG GC) into pMW46.

pAF44 – sgRNA: GTC GTT TCG AGA CAC AGC GG targeting N-terminus of *gei-17.* Cloned with OAF224 (cttg GTC GTT TCG AGA CAC AGC GG) and OAF225 (aaac CCG CTG TGT CTC GAA ACG AC) into pMW46.

pAF50 - Cloned by Gibson assembly. TIR-1 sequence was amplified with OAF297 (ATG CAA AAG AGA ATC GCC TTG TCG) and OAF298 (GAG TCC GTT GGT GGT GAT GAT TTG AC) from pLZ31. The pCFJ151 containing the *SL2::mCherry::unc-54 3`UTR* and the *egl-17* promoter was cloned in two fragments with OAF172 (gtc gtg tct tac cgg gtt gga ctc aag acg ata gtt acc gga taa g)/ OAF309 (GGC GAT TCT CTT TTG CAT GAG CTC GGT ACC CTC CAA GCA AG) and OAF169 (gag tcc aac ccg gta aga cac gac)/ OAF308 (CAT CAC CAC CAA CGG ACT CTA Agc tgt ctc atc cta ctt tca cc) from pAF43.

pAF52 – Cloned by Gibson assembly. The *bar-1* promoter was amplified with OAF314 (GTA CCA GAG CTC ACC TAG GCT TAG CAA AGC CGT GTC AAA ACC CAC) and OAF316 (GGC GAT TCT CTT TTG CAT TTT TTA GCC TGC TTT TTT GTA CAA ACT TGC CC) from pEH5. The backbone containing the *TIR-1::SL2*::*mCherry*::*unc-54 3`UTR* was amplified in two fragments with OAF297 (ATG CAA AAG AGA ATC GCC TTG TCG)/ OAF169 (gag tcc aac ccg gta aga cac gac) and OAF172 (gtc gtg tct tac cgg gtt gga ctc aag acg ata gtt acc gga taa g)/ OAF315 (CCT AGG TGA GCT CTG GTA CCC TCT AG) from pAF50.

pAF55- pCFJ151 containing cdh-3p>TIR1::SL2::mCherry::unc-54 3`UTR. The *cdh-3* promoter was cloned with OAF330 (GTA CCA GAG CTC ACC TAG GTA GAG CAT GAT GTC CTT ACC TTG) and OAF321 (GGC GAT TCT CTT TTG CAT GAG CTC GGT ACC CTC CAA GCA AGG G) from pAF54 and OAF297 (ATG CAA AAG AGA ATC GCC TTG TCG)/ OAF169 (gag tcc aac ccg gta aga cac gac), OAF172 (gtc gtg tct tac cgg gtt gga ctc aag acg ata gtt acc gga taa g)/ OAF315 (CCT AGG TGA GCT CTG GTA CCC TCT AG) from pAF52.

pAF57 - The 5`homology arm was amplified from genomic DNA with OAF239 (CGA CGG CCA GTC GCC GGC AGC GTG AAC ATG CAC ACT CTG C) and OAF344 (GGC TGG ATC TTT AGG CAT CGT TTA TAG CGG GAG TCT CTG AAA C). The 3`homology arm was amplified with OAF345 (GGA TGA CGA TGA CAA GAG AAT GGC CGA TGA TGC AGC TCA AGC AGG) and OAF346 (GAC CAT GTT ATC GAT TTC CGA ATC TCG TGT CTC TGA TTT CTT GTC). Degron sequence was cloned from pLZ29 with OAF334 (ATG CCT AAA GAT CCA GCC AAA CCT C) and OAF335 (CTT CAC GAA CGC CGC CGC CTC CGG G). The backbone of plasmid containing the Self-Excising Selection Cassette was amplified in two fragments with OAF339 (CGG CGG CGT TCG TGA AGG AGA ATC TGT ACT TTC AAT CCG GAA AG)/ OAF340 (CAT TCT CTT GTC ATC GTC ATC CTT G) and OAF343 (GGA AAT CGA TAA CAT GGT CAT AGC TGT TTC C)/ OAF337 (CTG CCG GCG ACT GGC CGT CGT TTT ACA AC) from pDD282. A point mutation in the right homology arm was corrected with OAF347 (GTC CTC TAC CTA CAC TCT AAT CCG)/ OAF337 (CTG CCG GCG ACT GGC CGT CGT TTT ACA AC) and OAF239 (CGA CGG CCA GTC GCC GGC AGC GTG AAC ATG CAC ACT CTG C)/ OAF348 (GTG TAG GTA GAG GAC TAG AAT CCG CCC). Cloned by Gibson assembly.

pAF58 - The 5`homology arm was amplified from genomic DNA with OAF336 (GAC GGC CAG TCG CCG GCA GGA GAT CAC GAC GCA CTT TAT AG) and OAF338 (GGC TGG ATC TTT AGG CAT ATT GAT TTC AAT ACT TCC GAT TTT CCT CTG). The 3`homology arm was amplified with OAF341 (GGA TGA CGA TGA CAA GAG AAT GTT ACC GAA TAA TCA ATG GCA AAT AC)/ OAF342 (GAC CAT GTT ATC GAT TTC CTC ATA ATT TCC GTT CGG GAA ATT GG). The backbone containing the Self-Excising Selection Cassette and degron sequence was cloned in two fragments with OAF334 (ATG CCT AAA GAT CCA GCC AAA CCT C)/ OAF340 (CAT TCT CTT GTC ATC GTC ATC CTT G) and OAF343 (GGA AAT CGA TAA CAT GGT CAT AGC TGT TTC C) OAF337 (CTG CCG GCG ACT GGC CGT CGT TTT ACA AC) from pAF56..

pAF59 – sgRNA targeting K10 in *lin-1* cloned with OAF369 (cttg GTC GAG TTC GGA AGA AGC CG) and OAF370 (aaac CGG CTT CTT CCG AAC TCG AC) into pMW46.

pAF62 – sgRNA targeting K169 in *lin-1* cloned with OAF375 (cttg GTT CAT ATT TGA GGA AAA GT) and OAF376 (aaac ACT TTT CCT CAA ATA TGA AC) into PMW46.

pAF63 - Cloned by Gibson assembly. The *hlh-2prox* promoter was amplified with OAF355 (CCA GAG CTC ACC TAG GGG ATC CTC ATC GTC TAC TTC TC) and OAF356 (CCA GAG CTC ACC TAG GGG ATC CTC ATC GTC TAC TTC TC) from pTD42. The backbone containing the *TIR-1::SL2*::*mCherry*::*unc-54 3`UTR* was amplified in three fragments with OAF357 (GCT TCA AGT TTG TAC AAA AAA GCA GGC)/ OAF249 (CCG TAC GTC TCG AGt gta aaa cga c) and OAF250 (gtt tta caC TCG AGA CGT ACG GTA AGT GCA AGT AAG ATC AG)/ OAF169 (gag tcc aac ccg gta aga cac gac) and OAF172 (gtc gtg tct tac cgg gtt gga ctc aag acg ata gtt acc gga taa g)/OAF315 (CCT AGG TGA GCT CTG GTA CCC TCT AG) from pAF52.
