# Supplementary material for: Tissue-specific inhibition of protein sumoylation uncovers diverse SUMO functions during C. elegans vulval development
Source: PLoS Genet. 2022 Jun 6;18(6):e1009978. doi: 10.1371/journal.pgen.1009978 (PMC9203017; doi:10.1371/journal.pgen.1009978)
Supplement: S3 Table — (DOCX) [file pgen.1009978.s006.docx]

**S3 Table. List of primers used**

| List of primers for genotyping: | | |
| --- | --- | --- |
| Name | Sequence 5`-3` | Used for: |
| OAF297 | ATG CAA AAG AGA ATC GCC TTG TCG | F primer to genotype TIR-1 |
| OAF298 | GAG TCC GTT GGT GGT GAT GAT TTG AC | R primer to genotype TIR-1 |
| OAF348 | GTG TAG GTA GAG GAC TAG AAT CCG CCC | F primer to genotype *smo-1(zh140)* |
| OAF349 | CTA CCG TAC CCT CGT GTT GCT CGT G | R primer to genotype *smo-1(zh140)* |
| OAF350 | GTA CGA GTA AGC CCC ACT TCA TTC | F primer to genotype *gei-17(zh142)* |
| OAF333 | CTC ACG TGT GCT GCC ATA TTT TCC TG | R primer to genotype *gei-17(zh142)* |
| OAF365 | CAG CTC GTC ACT CAT TGC CAC GTC | F primer to genotype *lin-1(zh157)* and *lin-1(159)* |
| OAF366 | GCC CAA TCA ATG CAC CAT GTC CCC | R primer to genotype *lin-1(zh157)* and *lin-1(159)* |
| OAF367 | GAT GCA CCA TGT CTT GCC GAC | F primer to genotype *lin-1(zh158)* and *lin-1(159)* |
| OAF368 | CCC AAA TCT TAC CTA TCT GTT GG | R primer to genotype *lin-1(zh158)* and *lin-1(159)* |
| ONG5 | TCG ACG GCG GCT AAT TAG CTT TCG C | allele-specific F primer to genotype K10A mutation in *lin-1* |
| ONG6 | TCG ACG GCG GCT AAT TAG CTT TTT T | allele-specific R primer to genotype K10A mutation in *lin-1* |
| List of primers for cloning: | | |
| OAF239 | CGA CGG CCA GTC GCC GGC AGC GTG AAC ATG CAC ACT CTG C | |
| OAF334 | ATG CCT AAA GAT CCA GCC AAA CCT C | |
| OAF335 | CTT CAC GAA CGC CGC CGC CTC CGG G | |
| OAF336 | GAC GGC CAG TCG CCG GCA GGA GAT CAC GAC GCA CTT TAT AG | |
| OAF337 | CTG CCG GCG ACT GGC CGT CGT TTT ACA AC | |
| OAF338 | GGC TGG ATC TTT AGG CAT ATT GAT TTC AAT ACT TCC GAT TTT CCT CTG | |
| OAF339 | CGG CGG CGT TCG TGA AGG AGA ATC TGT ACT TTC AAT CCG GAA AG | |
| OAF340 | CAT TCT CTT GTC ATC GTC ATC CTT G | |
| OAF341 | GGA TGA CGA TGA CAA GAG AAT GTT ACC GAA TAA TCA ATG GCA AAT AC | |
| OAF342 | GAC CAT GTT ATC GAT TTC CTC ATA ATT TCC GTT CGG GAA ATT GG | |
| OAF343 | GGA AAT CGA TAA CAT GGT CAT AGC TGT TTC C | |
| OAF344 | GGC TGG ATC TTT AGG CAT CGT TTA TAG CGG GAG TCT CTG AAA C | |
| OAF345 | GGA TGA CGA TGA CAA GAG AAT GGC CGA TGA TGC AGC TCA AGC AGG | |
| OAF346 | GAC CAT GTT ATC GAT TTC CGA ATC TCG TGT CTC TGA TTT CTT GTC | |
| OAF365 | CAG CTC GTC ACT CAT TGC CAC GTC | |
| OAF366 | GCC CAA TCA ATG CAC CAT GTC CCC | |
| OAF367 | GAT GCA CCA TGT CTT GCC GAC | |
| OAF368 | CCC AAA TCT TAC CTA TCT GTT GG | |
| ssDNA oligo for the repair template: | | |
| OAF377 | 5`GAA GGT TTG TCC ACT GAC TTC ATG ACT CGT AAA TAG TAT TCT TTT GCA GGT CGC GAA AGA GCC GCC GTC GAG TTC GGA AGA AGC CGA AGA AGA AGA ATC TCC GAA ACA TAC GAT TGA GGG 3` - PAGE purified oligonucleotide. | |
| OAF378 | 5` GTA ACT ACT GAC GCC CAC GCT CCG CCG ACC GCT GAC TTT TCC TCA AAT ATG AAC ATG AAG ATG TGT TAT GCC GCG GAC GAG AAA GAC ATT CGA CAC GAG ATT CCG TCG TTT ATG ACG TCA TTA CA 3` - PAGE purified oligonucleotide. | |
