# Supplementary material for: Tissue-specific inhibition of protein sumoylation uncovers diverse SUMO functions during C. elegans vulval development
Source: PLoS Genet. 2022 Jun 6;18(6):e1009978. doi: 10.1371/journal.pgen.1009978 (PMC9203017; doi:10.1371/journal.pgen.1009978)
Supplement: S4 Table — (DOCX) [file pgen.1009978.s007.docx]

**S4 Table. Number of scored animals in 3 independent replicates for Fig. S1A.**

| Strain; condition: | L1/2 | L2 | L2/3 | L3 | L3/4 |
| --- | --- | --- | --- | --- | --- |
| *eft-3p>tir-1*; -IAA | 239 | 196 | 211 | 175 | 83 |
| *eft-3p>tir-1*; +IAA | 213 | 151 | 186 | 167 | 77 |
| *bar-1p>tir-1*; -IAA | 211 | 194 | 182 | 180 | 146 |
| *bar-1p>tir-1*; +IAA | 153 | 166 | 171 | 162 | 99 |
