# Supplementary material for: Tissue-specific inhibition of protein sumoylation uncovers diverse SUMO functions during C. elegans vulval development
Source: PLoS Genet. 2022 Jun 6;18(6):e1009978. doi: 10.1371/journal.pgen.1009978 (PMC9203017; doi:10.1371/journal.pgen.1009978)
Supplement: S5 Table — (DOCX) [file pgen.1009978.s008.docx]

**S5 Table. Number of animals scored in 3 independent replicates for Fig. S1B.**

| Strain; condition: | L1/2 | L2 | L3 | L3/4 | L4 |
| --- | --- | --- | --- | --- | --- |
| *eft-3p>tir-1;* -IAA | 64 | 73 | 59 | 67 | 56 |
| *eft-3p>tir-1;* +IAA | 79 | 59 | 51 | 65 | 49 |
| *bar-1p>tir-1;* -IAA | 107 | 94 | 61 | 65 | 65 |
| *bar-1p>tir-1;* +IAA | 64 | 74 | 39 | 49 | 37 |
