# Supplementary material for: Tissue-specific inhibition of protein sumoylation uncovers diverse SUMO functions during C. elegans vulval development
Source: PLoS Genet. 2022 Jun 6;18(6):e1009978. doi: 10.1371/journal.pgen.1009978 (PMC9203017; doi:10.1371/journal.pgen.1009978)
Supplement: Attachment — Submitted filename: point-by-point reply.pdf [file pgen.1009978.s010.pdf]

Point-by-point response

To Reviewer #1:

(1) I am not convinced that the manuscript is within the exact scope of PLOS Genetics. The novelty of this work is primarily in applying the AID system for the study of the role of pleiotropic proteins in vivo. I think that a journal focusing on techniques and methods will be more suitable for this type of paper.

The reviewer is correct in the sense that the AID system is well established in *C. elegans* and we do not add to the general method. However, we believe the novelty of our manuscript is in the way we use the AID system to dissect the various roles of the SUMO pathway in a developmental process, using the C. elegans vulva as a model for organogenesis. To our knowledge, the approach to manipulate the SUMO pathway in a tissue-specific and temporally controlled fashion is a unique way to dissect the pleiotropic effects of protein sumoylation down to individual phenotypes in different tissues (**lines 316f**). Moreover, we provide direct evidence for the physiological significance of LIN-1 ETS sumoylation during epithelial morphogenesis. Sumoylation of various mammalian ETS family transcription factors has been reported, suggesting that this may be a commonly used mechanism to control their transcriptional activity (**lines 420f**). Our results on the *C. elegans* vulva demonstrate the significance of this protein modification at a cellular level. Finally, we show that one specific sumoylation site (K169 in LIN-1) affects one distinct phenotype- ventral toroid contraction.

(2) There is no biochemical evidence for the activity, effectiveness, and specificity of the system. One major gold standard of the SUMO field is to show biochemically that a protein claimed to be SUMOylated is indeed SUMOylated in vivo. In this manuscript, there is no biochemical evidence want so ever for the activity of the system.

AND

Morphological analysis can hint at the process affected but provides only the end result of the effect. Measuring the GFP levels of target proteins does not demonstrate directly that these proteins are SUMOylated in wild-type conditions and not SUMOylated when the AID system is active. The cehnges in the levels/ position of the GFP signal may stem from the activity of SUMO on a regulator of the proposed target gene. I would strongly recommend showing biochemically that the system can change the SUMOlyation state of at least one target protein.

This is a critical point as we do not provide direct biochemical evidence for the sumoylation of LIN-1 in the VPCs (**lines 407f**). Detecting LIN-1 sumoylation biochemically in total worm extracts has not been possible due to the very low expression levels, even when using the endogenously tagged allele. This is probably also the reason, why it has not been possible to detect LIN-1 sumoylation by proteomic analysis of whole worm extracts (Kaminsky et al 2009 and Drabikowski et al 2018). Also, since the endogenous LIN-1 reporter is most strongly expressed in the VPCs and only very faintly in surrounding tissues during larval development, it is possible that LIN-1 sumoylation occurs predominantly in the VPCs, which would make it even more difficult to detect.

As an alternative, we have quantified free SUMO levels by Western blot analysis and shown that AID::SMO-1 levels are strongly reduced (by 95%) after AID degradation (**lines 130-136** & **new Fig. 2**). It is therefore very likely that our approach results in a strong reduction in the sumoylation of all targets protein in the cells, in which the AID system is activated. Biochemical analysis cannot identify the cells or tissues, in which sumoylation of a specific target such as LIN-1 occurs. Our indirect assay quantifying LIN-1 levels after AID::SMO-1 mediated-degradation is to date the only evidence indicating that LIN-1 is indeed sumoylated in the VPCs (**lines 405f**). The use of LIN-1 alleles with mutated SUMO sites is the best possible control for this assay, as it does exclude indirect effects. Taken together, we believe, the approach presented here is a valuable addition to the existing biochemical tools to study the SUMO pathway in individual tissues of an intact animal.

(3) The AID system is a well-established system used in C. elegans for a couple of years now for spatiotemporal knockdown of protein of interest. Moreover, the role of SUMO in the development of C. elegans vulva is quite well-characterized starting from 2004. I am not convinced that the manuscript really adds enough new data to justify a publication in PLOS genetics. The novelty of this manuscript is in combining the AID system with careful morphological and expression studies but the biological significance of the manuscripts remains in the realm of a proof of principle

It is correct that the role of the SUMO pathway in the *C. elegans* vulva has been studied before, mainly in the context of fate specification- albeit not in a tissue-specific manner via AID. In addition, we have identified several additional phenotypes and examined the tissues involved. Furthermore, by creating sumoylation-deficient *lin-1* alleles we can attribute one specific class of phenotypes (toroid contraction) to a single SUMO site in this transcription factor. This is a new finding that demonstrates the precision one can achieve by applying the tissue-specific AID system (**lines 415**).

To Reviewer #2:

In this study the authors used the auxin-inducible protein degradation system to inhibit sumoylation either in the C. elegans anchor cell (AC) or the vulva. Four different promoters regulating the TIR-1 ubiquitin E3 ligases were used to inhibit the SUMO system before and after AC specification, in the primary VPCs and in all VPCs. In addition, a somatic promoter was used to degrade SUMO in somatic tissues. This study proved that the TIR-1 system is working in the vulva, but new findings are limited as the specific drivers did not resulted in phenotypes therefore could not dissect the possible different functions and targets of SUMO in P6.p and in P5.p and P7.p and also the degradation was not enough or is not required in the AC.

See also above in our reply to reviewer #1 to a similar comment made under point (2)

The drivers used did not allow us to distinguish between 2° and 1° VPCs. We can only distinguish between VPCs, AC and other tissues. Though, the analysis shown in **Figs 1 and 2** indicated that the degradation by AID is specific and very efficient, both in the VPCs and AC. Some of the phenotypes, especially in the AC and during toroid morphogenesis have not been reported previously. Moreover, we could attribute one specific class of phenotypes -

toroid morphogenesis- to a single sumoylation site in the LIN-1 transcription factor (**lines 415**). This degree of specificity in the phenotypic analysis has not been attained to date.

Specific comments

1. Figure 1B: the insets could be bigger as it is difficult to see the degradation of the GFP happening with the small insets. Also, the insets could be shown for every promoter rather than just the first promoter.

Figure 1B has been improved with larger insets for all drivers used.

2. Figure 2C. The data on AID::SUMO is not shown.?

Vulval induction counts for AID::SMO-1 ± auxin with the VPC-specific *tir-1* driver are shown in lines 3 and 4 of the table in **Fig. 3C** (formerly Fig. 2C). We are not sure what additional data could be missing.

3. Figure 2C. SUMO degradation cause ectopic posterior vulva while GEI-17 did not, did the authors suspect specific activity of another sumo ligase?

Overall, degradation of SMO-1 caused more severe phenotypes than GEI-17 degradation. This could be due to another E3 ligase besides GEI-17, to sumoylation occurring independently of and E3 ligase or to the degradation of AID::SMO-1 tagged target proteins. These possibilities are discussed on **lines 51, 210, 338ff**.

4. LIN-1 - what was the reasoning to change K to alanine residues and not to arginine? Maybe LIN-1 was destabilized as the charge was changed and another option is that the auxin system could be leaky specifically on this target.

We considered the modification to an uncharged alanine to be the “safer” option to prevent potential leakiness that may occur after replacement with an arginine. Moreover, Leight et al. 2005 have shown that the K to A mutations efficiently block LIN-1 sumoylation in vitro. We now discuss the possibilty that the mutations could have a destabilizing effect on LIN-1, or affect other post-translational modifications (**line 280f**).

5. There is no evidence in the data presented that substantial fraction of endogenous LIN-1 is sumoylated in the 1° VPC and that degradation of SUMO lead to degradation of its substrates.

We cannot say what fraction of LIN-1 is sumoylated in the VPCs at any given time point. But, due to the transient and reversible nature of the SUMO modification, even a small fraction of LIN-1 sumoylation will result in a progressive loss of LIN-1 expression over time through repetitive cycles of LIN-1 modification with AID::SMO-1 followed by proteasomal degradation. This is now discussed on **line 350ff.** The fact that mutations of the SUMO sites render LIN-1 insensitive to AID::SMO-1 degradation is good evidence for the specificity of the targeted degradation as opposed to indirect effects of the SUMO pathway on LIN-1 levels (**line 269 & 276f**).

Additional comments:
Line 50- sumo is attached to substrates also without E3 ligase (in contrast to ubiquitin)

This is now mentioned in the introduction and discussion (**lines 51** and **340**)

Line 62- comment - corrected

Line 82- it is hard to hypothesize this as sumoylation is transient and only a small fraction of a substrate is sumoylated. This system is expected to degrade free SUMO, this will cause a decrease in sumoylation of targets.

AID::SMO-1 likely degrades both free SUMO (as shown in the new data in **Fig. 2C,D**) as well as AID::SMO-1 modified target proteins, once the animals are exposed to auxin. As discussed above, due to the transient nature of the modification and the rapid cycling through sumoylated and de-sumoylated states, even a low proportion of sumoylation is expected to lead to the progressive degradation of target proteins (**line 350f**).

To Reviewer #3:

Given the broad scope of this manuscript, I have only two recommendations to the authors. Given that vulva papers are rare these days, the manuscript would profit from two philosophical additions in the introduction and the discussion:

1. In the introduction, I would urge the authors to add a more detailed description of pleiotropy and redundancy as general principles in genetics. Vulva development in C. elegans provides some of the best examples in all of animal development (besides Drosophila). I find it an important point to highlight that what the authors call 'complexity' is in reality, in large parts, the combination of pleiotropy and redundancy of several genes and signaling pathways involved in the regulation of vulva development. As this manuscript will likely be the most modern on vulva development for some time, this addition might be important.

This is a good point. The *C. elegans* vulva is an ideal model to dissect the pleiotropic effects of the SUMO pathway. This was one of the reasons we applied the tissue-specific approach to study the effects on vulval development. We are now mentioning this point in the introduction (**line 58**), the abstract (**line 15**) and at the beginning of the discussion (**line 305**), albeit briefly due to space constraints.

2. In the Discussion, the manuscript would profit from the addition of an evolutionary interpretation of the authors findings; both, with regard to the evolution of vulva development and the evolution of the role of sumoylation in animal development.

We believe, the most relevant evolutionary aspect is the observation that many mammalian ETS transcription factors, including human ETS-1, have been reported to be SUMO targets. It thus appears, that like phosphorylation, sumoylation of ETS family transcription factors is a commonly used mechanism to control their transcriptional activity. We now make this point in the concluding paragraph of the discussion (**line 419f**)

To Reviewer #4:

Major comments
1. I would like to see a concise summary of the tissues/cells examined and the effects to make it easier for the non-vulval specialist to comprehend how the pieces fit together. It might be good as a table with cell type, phenotype, and whether affected.

This is a good suggestion. We are now including **Fig. 6** to summarize our findings by showing which tissues contribute to the different phenotypes.

2. Some effects might be independent or downstream knock-on effects of a primary perturbation especially since there is extensive cross-talk among the targeted cell types/stages. Specifically, what is the minimum set of independent effects you see and what might be the maximum. Are all phenotypes autonomous (based on existing knowledge in the field) This can be set in the context of the summary I requested in point 1.

We think the minimum/most specific independent effect is the failure of the toroids to contract, as we could attribute this to a single SUMO site on LIN-1. The maximum is difficult to answer, as for example the defects in vulval fate specification could be a combination of transcriptional changes in the VPCs and the mispositioning of the AC, which secretes EGF to induce the 1° VPC fate. There are clearly non-autonomous defects, as mentioned on **line 230** and in the discussion on **lines 335** & **356f**. **Fig. 6** should also illustrate the cell autonomy versus non-autonomy of the different phenotypes.

minor comment
The specific choice of references seems odd. I think they are overall fine, but I just wanted to make sure the choices of which specific primary articles and reviews to cite were conscious and not random.

Since we are certainly not experts on the vertebrate SUMO pathway, we are citing mainly the review articles that we found useful to obtain an overview of the vertebrate SUMO data. We are, however, confident that we are citing all the relevant papers from the *C. elegans* field.
